# A UPLC Q-Exactive Orbitrap Mass Spectrometry-Based Metabolomic Study of Serum and Tumor Tissue in Patients with Papillary Thyroid Cancer

**DOI:** 10.3390/toxics11010044

**Published:** 2022-12-31

**Authors:** Bo Xu, Wei Gao, Ting Xu, Cuiping Liu, Dan Wu, Wei Tang

**Affiliations:** 1 State Key Laboratory of Reproductive Medicine, School of Public Health, Nanjing Medical University, Nanjing 211166, China; 2 Department of Endocrinology, Nanjing Medical University Affiliated Geriatric Hospital, Nanjing 210024, China; 3 Bank of Biological Samples, First Affiliated Hospital of Nanjing Medical University, Nanjing 210029, China

**Keywords:** papillary thyroid cancer, metabolomics, lymph node metastasis, tumor tissue, serum

## Abstract

Objective: To find the metabolomic characteristics of tumor or para-tumor tissues, and the differences in serums from papillary thyroid cancer (PTC) patients with or without lymph node metastasis. Methods: We collected serums of PTC patients with/without lymph node metastasis (SN1/SN0), tumor and adjacent tumor tissues of PTC patients with lymph node metastasis (TN1 and PN1), and without lymph node metastasis (TN0 and PN0). Metabolite detection was performed by ultra-high performance liquid chromatography combined with Q-Exactive orbitrap mass spectrometry (UPLC Q-Exactive). Results: There were 31, 15, differential metabolites in the comparisons of TN1 and PN1, TN0 and PN0, respectively. Seven uniquely increased metabolites and fourteen uniquely decreased metabolites appeared in the lymph node metastasis (TN1 and PN1) group. Meanwhile, the results indicated that four pathways were co-owned pathways in two comparisons (TN1 and PN1, TN0 and PN0), and four unique pathways presented in the lymph node metastasis (TN1 and PN1) group. Conclusions: Common or differential metabolites and metabolic pathways were detected in the lymph node metastasis and non-metastatic group, which might provide novel ways for the diagnosis and treatment of PTC.

## 1. Introduction

Thyroid cancer is the most common endocrine malignancy disease. Papillary thyroid cancer (PTC), the major subtype, is derived from thyroid follicular cells [1]. The 30-year recurrence rate (29.4%) and cause-specific mortality (8.6%) of PTC still raise concern [2]. As PTC is most likely to metastasize to cervical lymph nodes in 30% to 90% of patients [3,4], lymph node metastasis is a critical risk factor for PTC recurrence and distant metastasis [5]. At present, ultrasound is the most commonly used diagnostic method for thyroid cancer [6,7,8,9]. The sensitivity and specificity of ultrasound detection in predicting central lymph node metastasis of PTC ranged from 40.4% to 92.9% and from 39.7% to 79.4%, respectively [10]. In addition, there is still no consensus on the supplementary diagnosis markers; for example, the predictive value of HBME-1 in lymph node metastasis of PTC is still conflict [11,12], and the mutations of BRAF and TERT promoter in predicting lymph node metastasis of PTC are still controversial [10,13,14,15,16,17]. Therefore, it is necessary to seek new markers of PTC with lymph node metastasis. Metabolic change is one of the important markers of tumors [18]. Metabolomics is an analytical study of multiple low-molecular-weight metabolites, and it is also downstream of gene expression and protein activity [19]. At the moment, metabolomics is widely used in disease prediction, mechanism exploration, and for providing new diagnostic molecular markers and therapeutic targets [20,21].

The metabolomics of PTC have been studied by gas chromatography–mass spectrometry (GC–MS) [22] and nuclear magnetic resonance (NMR) [23]. However, different analytical platforms have different metabolic coverage, which can provide various metabolomics information [24]. Liquid chromatography–mass spectrometry (LC–MS) is a commonly used technology in metabolomics analysis with high sensitivity and broad coverage [25]. There were insufficient published metabolomic studies on PTC using LC–MS. In addition, the understanding of metabolomics alterations with respect to lymph node metastasis is still deficient. Given these facts, it is necessary to adopt LC–MS-based metabolomics technology to observe the detailed metabolic changes and disordered metabolic pathways in PTC patients with lymph node metastasis.

Therefore, we performed the current study using an ultra-high performance liquid chromatography tandem Q-Exactive mass spectrometry (UPLC Q-Exactive MS) to detect the differences in serum and tumor tissue metabolism profiles between PTC patients with and without lymph node metastasis, looking for metabolic markers that could serve as diagnostic and prognostic indicators for PTC and new therapeutic targets for PTC.

## 2. Materials and Methods

### 2.1. Subjects Recruitment

This study was approved by the Ethics Committee of the First Affiliated Hospital of Nanjing Medical University. All the participants signed the informed consent forms. All the participants had undergone thyroidectomy between February 2013 and May 2017. Histological assessment was carried out according to the criteria established by the World Health Organization [26]. Pathological diagnosis was performed independently by two pathologists.

We set inclusion criteria as following: (1) patients who were 18 to 65 years old; (2) patients without distant metastasis. There were 34 patients that provided both tissue samples and serum samples. We excluded patients with other malignant tumors, hypertension, diabetes, thyroid dysfunction or other diseases which might influence metabolism, and patients who had long-term use of drugs. A total of 12 patients were excluded, including 2 patients with diabetes, 5 patients with hypertension, 2 patients with diabetes and hypertension, 2 patients with thyroid dysfunction and 1 patient with other malignant tumor.

Finally, there were 8 patients with lymph node metastasis that provided tumor tissues (TN1), para-tumor tissues (PN1) and blood serum (SN1). For patients without lymph node metastasis, there were 14 individuals that provided tumor tissues (TN0), para-tumor tissues (PN0) and blood serum (SN0). The workflow for this study is shown in Figure S1.

### 2.2. Metabolomic Analysis

Samples were prepared according to the previous literature [27]. The tissue preparation steps were as follows: 50 mg of frozen tissue was cut with surgical scissors, then mixed with 750 μL ultra-pure water and ultrasonicated (power: 60%). The supernatant was obtained after centrifugation (16,000× *g*, 15 min, 4 °C). Then, internal standards were added. The dried residues were reconstituted for metabolomic analysis. Serum sample preparation steps were as follows: 40 μL methanol and internal standards were added into 10 μL of serum. After centrifugation (16,000× *g*, 15 min, 4 °C), the supernatant was obtained. Then, the dried residues were reconstituted for metabolomic analysis.

The metabolomic analysis was performed as a previous report [27]. Briefly, UPLC Q-Exactive MS analysis was performed using a UPLC Ultimate 3000 system (Dionex, Germering, Germany) plus a Q-Exactive mass spectrometer (Thermo Fisher Scientific, Bremen, Germany) in both positive and negative modes with fullscan acquisition (70–1050 *m/z*). The instrument performed at 70,000 resolution. A multistep gradient (mobile phase A: 0.1% formic acid in ultra-pure water, mobile phase B: 0.1% formic acid in pure CAN) was operated at a flow rate of 0.4 mL/min, and the runtime was 15 min. The metabolite identification was based on the comparisons of retention time and accurate mass with metabolite standards. All samples were analyzed in a randomized manner to avoid effects induced by the injection order.

### 2.3. Statistical Analysis and Bioinformatics Analysis

Data collation was performed with Excel. Data analysis was performed with IBM SPSS Statistics Premium V25.0 and R. Patients’ clinical information between groups was analyzed with a t test and Fisher’s exact test. According to the relative quantification of metabolites, differential metabolites were screened with a t-test after log transformation. Metabolite pathway and enrichment analysis were performed using MetaboAnalyst 5.0. Statistical significance was defined using a Benjamini-Hochberg (B-H) FDR <0.05. A visualization of altered metabolites in the global metabolic network was constructed with iPath 3.0. A Venn diagram was made with jvenn.

## 3. Results

### 3.1. Population Characteristics

The tissue and serum samples (n = 22, male/female: 3/19, metastasis/no metastasis: 8/14) of PTC patients were collected. The clinical information of each group was presented in Table S1. No significant differences in gender, age and body mass index were found. The difference in maximum tumor diameter (cm) between the two groups was statistically significant, which revealed that the size of PTC tumors affects the lymph node metastasis of PTC to some extent. Our result was consistent with a previous study [28].

### 3.2. The Altered Metabolites

Finally, a total of 152 metabolites and 129 metabolites were annotated from the detected spectral features from UPLC Q-Exactive in tissues and serums respectively. In total, 31 metabolites were significantly altered (*p* < 0.05 and FDR < 0.05) in the comparison between TN1 and PN1. Among these altered metabolites, 23 metabolites decreased and 8 metabolites increased (Table 1). We found 15 metabolites were significantly altered (*p* < 0.05 and FDR < 0.05) in the comparison between TN0 and PN0. When compared to the PN0 group, 13 metabolites were significantly down-regulated, while 2 metabolites were significantly up-regulated in the TN0 group (Table 2). We identified two increased metabolites and two decreased metabolites in the SN1 group compared with the SN0 group (*p* < 0.05), while there was no altered metabolite found after FDR calibration (Table 3).

### 3.3. The Co-Owned Metabolic Changes

We found nine significantly decreased metabolites and one significantly increased metabolite in two comparisons (TN1 and PN1, TN0 and PN0). 

### 3.4. Differential Metabolites between Two Comparisons (TN1 and PN1, TN0 and PN0)

**Figure 1:**
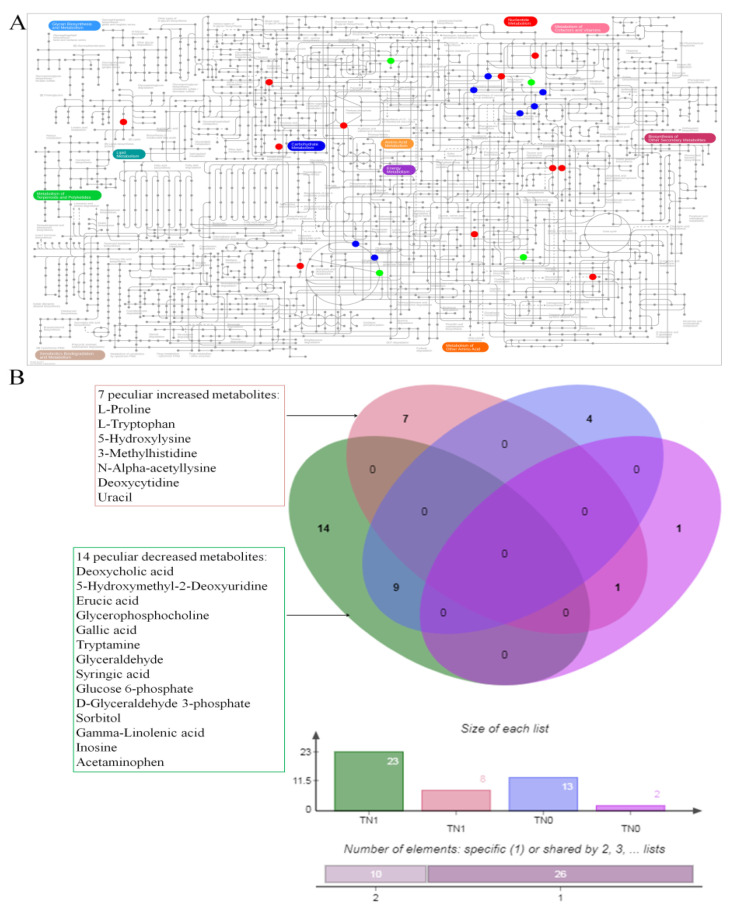
The altered metabolites. (**A**) Visualization of the altered metabolite coverage based on iPath 3.0. The blue points represent the co-owned metabolites in two groups (TN1 and PN1, TN0 and PN0), the red points represent the specific metabolites detected in the lymph node metastasis (TN1 and PN1) group, and the green points represent the specific metabolites detected in the group without lymph node metastasis (TN0 and PN0). The metabolites which are not indicated in the general pathway map are not shown. (**B**) This Venn diagram displays overlaps of differential metabolites between two groups (TN1 and PN1, TN0 and PN0).

We found that the PTC patients with lymph node metastasis (TN1 and PN1) had more differential metabolites than those without lymph node metastasis (TN0 and PN0). A visualization of the altered metabolic coverage based on iPath 3.0 is shown in Figure 1A. The detailed differential metabolites are as follows: 7 uniquely increased metabolites and 14 uniquely decreased metabolites appeared in the TN1 group when compared with the PN1 group (Figure 1B).

### 3.5. The Altered Pathways

**Figure 2:**
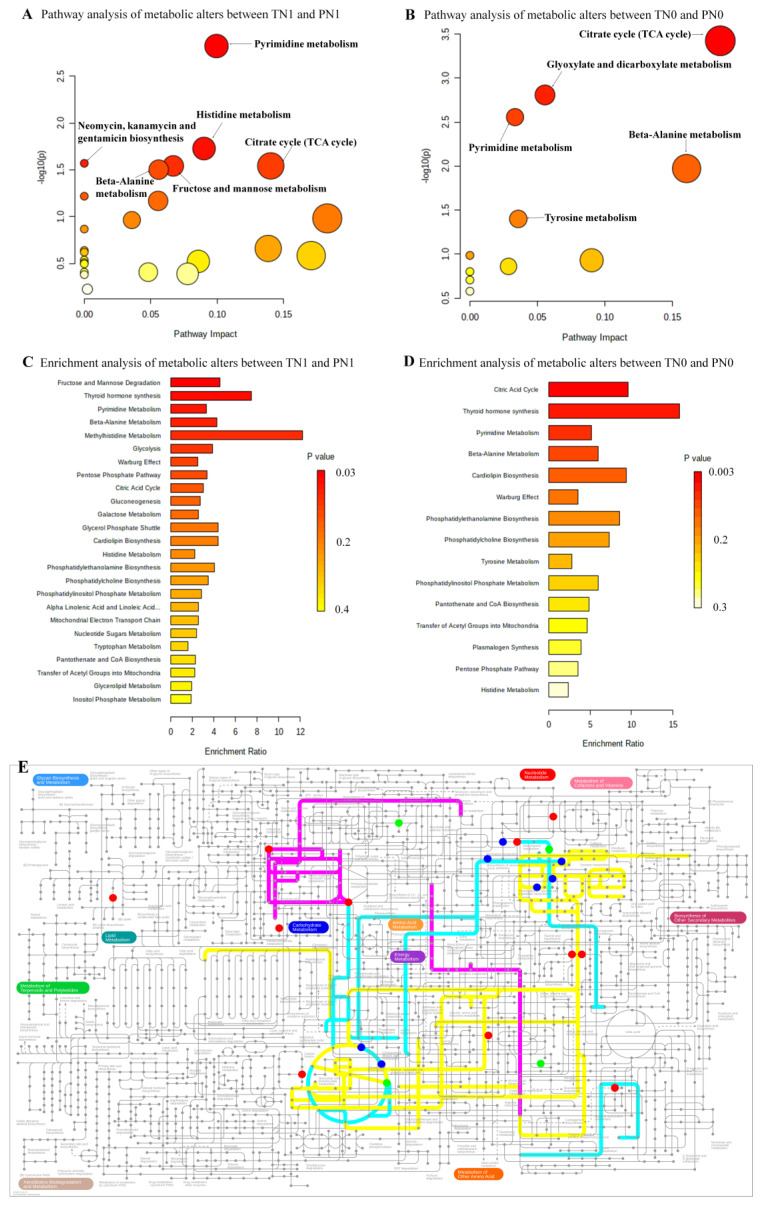
The altered pathways. (**A**) Pathway analysis of metabolic alterations between TN1 and PN1. (**B**) Pathway analysis of metabolic alterations between TN0 and PN0. (**C**) Enrichment analysis of metabolic alterations between TN1 and PN1. (**D**) Enrichment analysis of metabolic alterations between TN0 and PN0. (**E**) The metabolic network of the differential metabolites and altered metabolic pathways in the KEGG general metabolic pathway map. The cyan lines represent the co-owned pathways in two groups (TN1 and PN1, TN0 and PN0), the purple lines represent the specific metabolites detected in the lymph node metastasis (TN1 and PN1) group, and the yellow lines represent the specific metabolites detected in the group without lymph node metastasis (TN0 and PN0). The metabolites which are not indicated in the general pathway map are not shown.

Metabolic pathway analysis and enrichment analysis of differential metabolites between TN1 and PN1 showed that there were statistical differences (*p* < 0.05 and FDR < 0.05) in pyrimidine metabolism, histidine metabolism, neomycin, kanamycin and gentamicin biosynthesis, fructose and mannose metabolism, citrate cycle (TCA cycle), beta-alanine metabolism, fructose and mannose degradation and thyroid hormone synthesis. Compared with PN0, there were six significantly altered (*p* < 0.05 and FDR < 0.05) pathways in TN0 as follows: citrate cycle (TCA cycle), glyoxylate and dicarboxylate metabolism, pyrimidine metabolism, beta-alanine metabolism, tyrosine metabolism and thyroid hormone synthesis (Figure 2, Table S2). Our results indicated that four pathways (pyrimidine metabolism, beta-alanine metabolism, thyroid hormone synthesis and citrate cycle) were co-owned pathways in the comparisons between TN1 and PN1, and between TN0 and PN0. On the other hand, we identified four unique pathways (histidine metabolism, neomycin, kanamycin and gentamicin biosynthesis, fructose and mannose metabolism, fructose and mannose degradation) presented in the TN1 group when compared with the PN1 group, and two unique pathways (glyoxylate and dicarboxylate metabolism, tyrosine metabolism) presented in the TN0 group when compared with the PN0 group (Figure 2E).

## 4. Discussion

Our UPLC-HRMS study examined both serums and tissues of PTC patients with or without lymph node metastasis, and we found co-owned and differential metabolites/metabolite pathways.

First of all, in the comparisons (TN1 and PN1, TN0 and PN0), we found nine co-owned decreased metabolites (thyroxine, allantoin, iodotyrosine, cytidine monophosphate, cis-aconitic acid, citric acid, uridine, glycolic acid, rhamnose) and one co-owned increased metabolite (carnosine). Among these metabolites, allantoin has been implicated in prostate, colon, intestinal ovarian and breast cancer protection [29], and decreased citric acid has been found in prostate cancer [30]. Thyroxine is a hormone made in the thyroid gland. A large population study showed that L-thyroxine treatment was associated with a decreased frequency of PTC, so the decreased thyroxine of PTC in our study is reasonable. Our results indicated that the ability of the thyroid to synthesize thyroxine is affected after carcinogenesis [31]. Carnosine is a low-molecular-weight hydrophilic antioxidant, and it is important for many normal body functions. It has been suggested that carnosine may not display the same function in different tissues and may even play several functions within one tissue [32]. Although some studies have shown that carnosine has a positive effect on disease treatment [33], carnosine can also exert negative effects under certain conditions [34]. Our results suggest that these metabolites might be important metabolite markers of PTC.

Furthermore, we found 7 uniquely increased metabolites (L-proline, L-tryptophan, 5-hydroxylysine, 3-methylhistidine, N-alpha-acetyllysine, deoxycytidine, uracil) and 14 uniquely decreased metabolites (deoxycholic acid, 5-hydroxymethyl-2-deoxyuridine, erucic acid, glycerophosphocholine, gallic acid, tryptamine, glyceraldehyde, syringic acid, glucose 6-phosphate, D-glyceraldehyde 3-phosphate, Sorbitol, gamma-linolenic acid, inosine, acetaminophen) in the lymph node metastasis (TN1 and PN1) group when compared with the group without lymph node metastasis (TN0 and PN0). Most of the seven uniquely increased metabolites have been verified to possibly promote the progression of other tumors; for example, L-proline addition and L-tryptophan metabolism promote tumor invasion and metastasis and accelerate cancer progression [35,36]. The concentrations of 5-hydroxylysine and 3-methylhistidine have been identified as significant prognostic factors for overall survival in oral squamous cell carcinoma [37]. We speculated that these metabolites might promote the lymph node metastasis of PTC. On the other hand, among the 14 uniquely decreased metabolites, most of them have been reported to possibly inhibit the progression of other cancers. For instance, deoxycholic acid, erucic acid and syringic acid act in an anti-tumoral way in human colorectal cancer cells, neuroblastoma/glioblastoma and gallbladder cancer separately [38,39,40]. A switch from high to low levels of glycerophosphocholine could induce ovarian tumor aggressiveness [41]. Gallic acid and sorbitol could induce apoptosis of human small cell lung cancer cells/human gastric adenocarcinoma cells and human colorectal cancer cells separately [42,43,44]. The intratumoral injection of tryptamine was certified to reduce tumor growth and tumor sizes in vivo [45]. Gamma-linolenic acid can inhibit the invasion of human colon cancer cells [46]. These metabolites might also have inhibitory effects on PTC metastasis; future studies are warranted to assess these biomarkers as candidate biomarkers.

Our results indicated that four pathways (pyrimidine metabolism, beta-alanine metabolism, thyroid hormone synthesis and citrate cycle) were co-owned pathways in the comparisons of TN1 and PN1, and TN0 and PN0. Pyrimidine metabolism is required for tumor cells to maintain high proliferation. A previous study showed that pyrimidine metabolism was significantly altered in PTC tumor tissues, which supported our results [47]. Beta-alanine metabolism, as well as pyrimidine metabolism, is a crucial process in carbohydrate metabolism. Consistent with our findings, the regulation of beta-alanine catabolism in cancer has been demonstrated in previous works [48,49]. Disturbance of thyroid hormone synthesis is the most common endocrine affliction. For thyroid hormone synthesis, an adequate supply of essential micronutrients to the thyroid gland is crucial [50]. Thyroid hormone synthesis is affected after carcinogenesis.. The consistent pathway changes also included the citric acid cycle. Our results showed that several molecules in the citric acid cycle were decreased, which indicated that citric acid cycle activity in PTC tumor tissues had decreased; therefore, the cancer cells might depend on high levels of aerobic glycolysis (the Warburg effect) as the major source for ATP to support cellular proliferation [51]. The disturbance of these pathways might be potential mechanisms of PTC.

As for the four unique pathways presented in the lymph node metastasis (TN1 and PN1) group, histidine metabolism has been shown to be an important pathway in distinguishing metastatic from extratumoral tissue in cutaneous melanoma, and can reflect carcinogenesis and cancer progression [52]. Mannose, closely related to fructose, is necessary for glycosylation [53]. Altered glycosylation is characteristic of aggressive cancers [54]. Therefore, histidine metabolism and fructose and mannose metabolism might be the possible mechanisms of the lymph node metastasis of PTC.

In short, our work may provide new clues for the underlying mechanisms regarding PTC with/without lymph node metastasis as well as potential therapeutic targets. However, the sample size of our study is small, and further studies are warranted in larger-scale populations.

## 5. Conclusions

This study found the distinct metabolites and metabolic pathways in PTC patients with/without lymph node metastasis. Co-owned metabolites presented in the comparisons (TN1 and PN1, TN0 and PN0) were potential markers of PTC. Unique metabolites presented in the lymph node metastasis group might be predictors and potential therapeutic targets of lymph node metastasis. The disturbance of pyrimidine metabolism, beta-alanine metabolism, thyroid hormone synthesis and citric acid cycle might be potential mechanisms of PTC. Unique pathways presented in the lymph node metastasis (TN1 and PN1) group might be the possible mechanisms of lymph node metastasis of PTC. Knowing these altered metabolites and metabolic pathways is helpful in determining therapeutic targets.

## Figures and Tables

**Table 1:** List of the altered metabolites identified in the comparison between TN1 and PN1.

Metabolites	Fold Change	*p*	FDR
Thyroxine	0.020	4.815 × 10^−3^	3.485 × 10^−2^
Deoxycholic acid	0.023	2.784 × 10^−3^	2.645 × 10^−2^
Allantoin	0.024	6.246 × 10^−3^	3.921 × 10^−2^
Iodotyrosine	0.027	4.068 × 10^−4^	1.030 × 10^−2^
5-Hydroxymethyl-2-Deoxyuridine	0.033	1.370 × 10^−5^	1.041 × 10^−3^
Erucic acid	0.041	1.027 × 10^−3^	1.562 × 10^−2^
Cytidine monophosphate	0.062	3.140 × 10^−3^	2.808 × 10^−2^
Cis-Aconitic acid	0.071	1.573 × 10^−3^	2.146 × 10^−2^
Citric acid	0.085	2.250 × 10^−5^	1.140 × 10^−3^
Glycerophosphocholine	0.150	6.354 × 10^−3^	3.921 × 10^−2^
Uridine	0.159	4.870 × 10^−5^	1.480 × 10^−3^
Glycolic acid	0.183	8.537 × 10^−4^	1.442 × 10^−2^
Gallic acid	0.242	2.610 × 10^−3^	2.645 × 10^−2^
Tryptamine	0.261	9.285 × 10^−3^	4.599 × 10^−2^
Glyceraldehyde	0.262	3.640 × 10^−5^	1.383 × 10^−3^
Syringic acid	0.267	4.436 × 10^−3^	3.393 × 10^−2^
Glucose 6-phosphate	0.269	8.055 × 10^−3^	4.535 × 10^−2^
D-Glyceraldehyde 3-phosphate	0.284	6.449 × 10^−3^	3.921 × 10^−2^
Sorbitol	0.320	4.972 × 10^−4^	1.080 × 10^−2^
Gamma-Linolenic acid	0.324	9.380 × 10^−3^	4.599 × 10^−2^
Rhamnose	0.332	6.122 × 10^−4^	1.163 × 10^−2^
Inosine	0.375	8.844 × 10^−3^	4.599 × 10^−2^
Acetaminophen	0.585	1.912 × 10^−3^	2.146 × 10^−2^
L-Proline	1.539	7.248 × 10^−3^	4.237 × 10^−2^
L-Tryptophan	1.747	4.464 × 10^−3^	3.393 × 10^−2^
5-Hydroxylysine	2.817	6.362 × 10^−3^	3.921 × 10^−2^
3-Methylhistidine	2.839	4.259 × 10^−3^	3.393 × 10^−2^
N-Alpha-acetyllysine	3.222	1.877 × 10^−3^	2.146 × 10^−2^
Deoxycytidine	3.236	8.773 × 10^−3^	4.599 × 10^−2^
Uracil	8.363	1.270 × 10^−5^	1.041 × 10^−3^
Carnosine	32.324	1.977 × 10^−3^	2.146 × 10^−2^

**Table 2:** List of the altered metabolites identified in comparison between TN0 and PN0.

Metablolites	Fold Change	*p*	FDR
cis-Aconitic acid	0.051	3.330 × 10^−5^	2.386 × 10^−3^
Allantoin	0.096	3.694 × 10^−4^	7.019 × 10^−3^
Iodotyrosine	0.128	1.420 × 10^−5^	2.158 × 10^−3^
Isocitric acid	0.144	2.484 × 10^−3^	2.696 × 10^−2^
Thyroxine	0.192	1.255 × 10^−3^	1.734 × 10^−2^
Cytidine monophosphate	0.216	7.922 × 10^−4^	1.204 × 10^−2^
Trizma Acetate	0.226	1.865 × 10^−4^	4.724 × 10^−3^
Uridine	0.259	4.710 × 10^−5^	2.386 × 10^−3^
Glycolic acid	0.274	2.966 × 10^−3^	3.005 × 10^−2^
Citric acid	0.299	3.094 × 10^−4^	6.718 × 10^−3^
Gluconolactone	0.329	1.368 × 10^−4^	4.724 × 10^−3^
N-Acetylglutamic acid	0.360	1.447 × 10^−3^	1.833 × 10^−2^
Rhamnose	0.378	7.166 × 10^−4^	1.204 × 10^−2^
Ureidopropionic acid	2.808	2.299 × 10^−3^	2.688 × 10^−2^
Carnosine	4.734	1.807 × 10^−4^	4.724 × 10^−3^

**Table 3:** List of the altered metabolites identified in the comparison between SN1 and SN0.

Metabolite Name	Fold Change	*p*	FDR
L-Malic acid	0.187	2.310 × 10^−2^	0.923
Thyroxine	0.649	2.102 × 10^−2^	0.923
Carnosine	1.605	4.933 × 10^−2^	0.923
Docosahexaenoic acid	1.632	4.614 × 10^−2^	0.923

## Data Availability

The data presented in this study are available on request from the corresponding author. The data are not publicly available due to privacy or ethical restrictions.

## Supplementary Materials

The following supporting information can be downloaded at: https://www.mdpi.com/article/10.3390/toxics11010044/s1, Figure S1: Strategy for the metabolomic study regarding PTC; Table S1: The clinical information of patients in different groups; Table S2. Altered pathways in comparisons (TN1 and PN1,TN0 and PN0) by pathway analysis and enrichment analysis.
