# Supplementary material for: A UPLC Q-Exactive Orbitrap Mass Spectrometry-Based Metabolomic Study of Serum and Tumor Tissue in Patients with Papillary Thyroid Cancer
Source: Toxics. 2022 Dec 31;11(1):44. doi: 10.3390/toxics11010044 (PMC9863332; doi:10.3390/toxics11010044)
Supplement: Supplementary file 1 [file toxics-11-00044-s001.zip › toxics-2105113-supplementary.pdf]

toxics

MDPI

Supplementary Materials

Table S1. The clinical information of patients in different groups.

	TN1/PN1/SN1	TN0/PN0/SN0	*p*
Male/female	1/7	2/12	0.709
Age	41.625 ± 8.733	41.714 ± 11.159	0.985
Body mass index	25.691 ± 3.027	24.086 ± 2.242	0.170
Maximum diameter of tumor (cm)	2.638 ± 0.774	1.857 ± 0.712	0.026

Table S2. Altered pathways in comparisons (TN1 and PN1,TN0 and PN0) by pathway analysis and enrichment analysis.

	Pathway	Total	Hits	*p*	FDR
TN1and PN1					
	Pathway analysis[a]				
	Pyrimidine metabolism	39	4	0.002	0.012
	Histidine metabolism	16	2	0.019	0.031
	Neomycin, kanamycin and gentamicin biosynthesis	2	1	0.027	0.031
	Fructose and mannose metabolism	20	2	0.029	0.031
	Citrate cycle (TCA cycle)	20	2	0.029	0.031
	Beta-Alanine metabolism	21	2	0.031	0.031
	Enrichment analysis[b]				
	Fructose and Mannose Degradation	32	3	0.025	0.030
	Thyroid hormone synthesis	13	2	0.027	0.030
	Pyrimidine Metabolism	59	4	0.029	0.030
	Beta-Alanine Metabolism	34	3	0.030	0.030
TN0 and PN0					
	Pathway analysis[a]				
	Citrate cycle (TCA cycle)	20	3	0.000	0.002
	Glyoxylate and dicarboxylate metabolism	32	3	0.002	0.005
	Pyrimidine metabolism	39	3	0.003	0.005
	Beta-Alanine metabolism	21	2	0.011	0.014
	Tyrosine metabolism	42	2	0.040	0.040
	Enrichment analysis[b]				
	Citric Acid Cycle	32	3	0.003	0.012
	Thyroid hormone synthesis	13	2	0.006	0.012
	Pyrimidine Metabolism	59	3	0.016	0.021
	Beta-Alanine Metabolism	34	2	0.041	0.041

[a] Pathway analysis of metabolic alters based on KEGG database.

b Enrichment analysis of metabolic alters based on SMPDB.

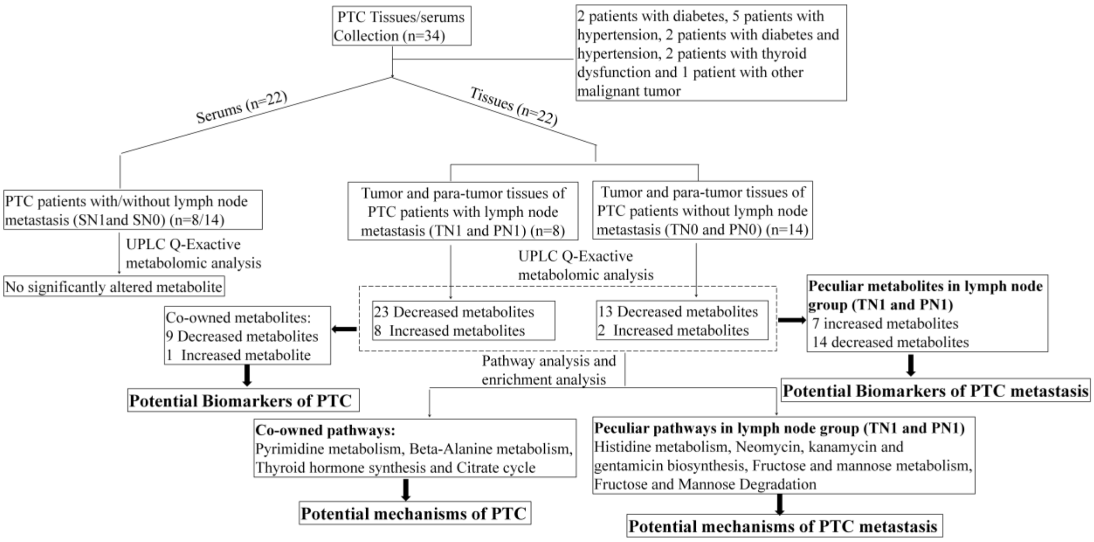

Figure S1. Strategy for the metabolomic study regarding PTC.
